# Protective Effect of Palm Oil-Derived Tocotrienol-Rich Fraction Against Retinal Neurodegenerative Changes in Rats with Streptozotocin-Induced Diabetic Retinopathy

**DOI:** 10.3390/biom10040556

**Published:** 2020-04-05

**Authors:** Muhammad Zulfiqah Sadikan, Nurul Alimah Abdul Nasir, Renu Agarwal, Nafeeza Mohd Ismail

**Affiliations:** 1 Centre for Neuroscience Research (NeuRon), Faculty of Medicine, Universiti Teknologi MARA, Sungai Buloh, Selangor 47000, Malaysia; mzsadikan@yahoo.com; 2 School of Medicine, International Medical University, Bukit Jalil, Kuala Lumpur 57000, Malaysia; RenuAgarwal@imu.edu.my (R.A.); NafeezaIsmail@imu.edu.my (N.M.I.)

**Keywords:** tocotrienol-rich fraction, diabetic retinopathy, angiogenesis, apoptosis, streptozotocin

## Abstract

Oxidative stress plays an important role in retinal neurodegeneration and angiogenesis associated with diabetes. In this study, we investigated the effect of the tocotrienol-rich fraction (TRF), a potent antioxidant, against diabetes-induced changes in retinal layer thickness (RLT), retinal cell count (RCC), retinal cell apoptosis, and retinal expression of vascular endothelial growth factor (VEGF) in rats. Additionally, the efficacy of TRF after administration by two different routes was compared. The diabetes was induced in Sprague-Dawley rats by intraperitoneal injection of streptozotocin. Subsequently, diabetic rats received either oral or topical treatment with vehicle or TRF. Additionally, a group of non-diabetic rats was included with either oral or topical treatment with a vehicle. After 12 weeks of the treatment period, rats were euthanized, and retinas were collected for measurement of RLT, RCC, retinal cell apoptosis, and VEGF expression. RLT and RCC in the ganglion cell layer were reduced in all diabetic groups compared to control groups (*p* < 0.01). However, at the end of the experimental period, oral TRF-treated rats showed a significantly greater RLT compared to topical TRF-treated rats. A similar observation was made for retinal cell apoptosis and VEGF expression. In conclusion, oral TRF supplementation protects against retinal degenerative changes and an increase in VEGF expression in rats with streptozotocin-induced diabetic retinopathy. Similar effects were not observed after topical administration of TRF.

## 1. Introduction

Diabetic retinopathy (DR) is a common microvascular complication associated with diabetes mellitus, which leads to vision impairment and blindness [1]. Globally, the approximate prevalence of DR among diabetic patients is 35%, and, among them, nearly 10% have the vision-threatening disease [2,3]. A strong positive correlation has been observed between chronic hyperglycemia and poor glycemic control with the development and progression of DR [4]. With the increasing annual incidence of diabetes mellitus [5,6], a higher number of DR cases are also expected in the future [7].

Retinal microvascular changes are the characteristic feature of DR; however, it has been observed that retinal neurodegeneration may appear even ahead of microvascular changes [8]. Diabetic retinal neurodegeneration involves retinal cell apoptosis, leading to thinning of retinal layers and loss of neuronal functions [9]. Hyperglycemia-induced oxidative stress, one of the important mechanisms involved in microvascular complications of diabetes mellitus [10], triggers cellular events that result in activation of various inflammatory cytokines and growth factors. These, in turn, accelerate neurodegenerative changes [11]. One of the important pathological features of DR is angiogenesis. Increased expression of vascular endothelial growth factor (VEGF), an angiogenic protein, is associated with diabetes-induced oxidative stress and neurodegeneration [12,13].

Palm oil-derived tocotrienol-rich fraction (TRF) consists of about 70% of tocotrienol and 30% tocopherol [14]. Both tocotrienol and tocopherol have four different isomers each: α, β, γ, and δ [15]. Tocotrienols have an unsaturated isoprenyl side chain, containing three double bonds at C-3′, C-7′, and C-11′ over a saturated phytyl side chain, making it different from tocopherols [16]. TRF acts as an antioxidant by scavenging free radicals through the hydrogen atom within the chromanol ring [17]. Antioxidants have been shown to be of therapeutic benefit in oxidative stress-induced diseases, including DR [18,19]. Since TRF has shown potent antioxidant effects in several human and animal studies [20,21,22,23], it may be of potential therapeutic value against DR. Additionally, some in vitro and in vivo studies have also shown that TRF produces anti-cancer effects due to its anti-angiogenic properties [24]. The anti-angiogenic properties of TRF have been attributed to its ability to suppress several signaling pathways, such as Ras-Raf-MEK-ERK and Fyn/HIF-1a pathways, leading to downregulation of VEGF expression [25]. Considering these beneficial properties of TRF, in this study, we investigated the effect of TRF on retinal morphology and expression of VEGF in a rat model of streptozotocin (STZ)-induced DR. Additionally, we compared the efficacy of orally administered TRF with that of topically administered TRF.

## 2. Materials and Methods

### 2.1. Animals

All experiments and animal handling were performed in compliance with the Associations for Research in Vision and Ophthalmology (ARVO) statement for the use of animals in ophthalmic and vision research, as well as local institutional ethical guidelines of Animal Care & Use Committee (ACUC) of Universiti Teknologi MARA under approval code UiTM CARE No: 286/2019. Male Sprague-Dawley rats, weighing 200–250 g, were obtained from Laboratory Animal Care Unit of Faculty of Medicine, Universiti Teknologi MARA. The animals were individually caged and maintained on a 12 h light/dark cycle with access to food and water ad libitum. All animals were subjected to one week of acclimatization and systemic and ophthalmic examination. Those found normal were included in the study.

### 2.2. Induction of Diabetes

To induce diabetes, rats were fasted overnight and then were administered with intraperitoneal (IP) injection of STZ, [2-Deoxy-2-(3-methyl-3-nitrosoureido)-D-glucopyranose (Santa Cruz Biotechnology Inc., Texas, USA) (STZ)], dissolved in ice-cold sodium citrate buffer (10 mmol/L, pH 4.5) at a single dose of 55 mg/kg body weight. Blood was collected from the tail vein, 48 h post-STZ injection for blood glucose estimation using Accu Chek Performa glucometer (Roche Diagnostic, Basel, Switzerland). Rats with a blood glucose level of more than 20 mmol/L were included for further study. The normal control group received an IP injection of sodium citrate buffer.

### 2.3. Topical Eye Drop Formulation of Tocotrienol-Rich Fraction

Tocotrienol-rich fraction (TRF) was supplied by ExcelVite Sdn Bhd, Perak, Malaysia (EVNol™ 50%, containing 12.3% α-tocopherol, 13.1% α-tocotrienol, 2.1% β-tocotrienol, 19.4% γ-tocotrienol, and 5.8% δ-tocotrienol). Topical eye drop formulation of 0.03% TRF was prepared, as described previously by Nasir et al. [26]. Empty formulation without TRF was used as a vehicle.

### 2.4. Study Design

Animals were divided into two groups based on the route of administration of treatment—oral treatment group and topical treatment group. Each of the two groups was further divided into three sub-groups. Three sub-groups of the oral treatment group consisted of nondiabetic rats treated with vehicle orally (NO), diabetic rats treated with vehicle orally (DVO), and diabetic rats treated with TRF orally (DTO). Similarly, 3 subgroups of topical treatment group consisted of nondiabetic rats treated with vehicle topically (NE), diabetic rats treated with vehicle topically (DVE), and diabetic rats treated with TRF topically (DTE). A total of 122 rats were included in the study, of which 32 rats were nondiabetic control rats and were divided into NO and NE (*n* = 16 each). The rest of the 90 rats were injected with STZ to induce diabetes. Among STZ-injected rats, 7 rats did not show an increase in the blood glucose above 20 mmol/L and were not included in further study. The rats that showed a blood glucose level above 20 mmol/L were considered diabetic and were randomly divided into DVO, DTO, DVE, and DTE. However, during the experimental period, 19 diabetic rats developed an infection and died. The rest of the 64 rats remained diabetic and survived the experimental period (*n* = 16 for each diabetic group). TRF was given orally in a dose of 100 mg/kg body weight [27] to rats in the oral treatment group (DTO). For topical application, 0.03% microemulsion formulation of TRF was used in DTE [26,28]. Olive oil was used as a vehicle for oral gavage (DVO), and empty formulation without TRF was used for topical administration (DVE).

Treatment by oral gavage was given once daily, whereas topical treatment was given in a volume of 10 μL, bilaterally, twice daily. All treatments were started 48 h post-STZ injection and were given for a period of 12 weeks. Blood glucose levels and body weight were monitored weekly during the experimental period. After 12 weeks of treatment, animals were sacrificed with an IP injection of sodium pentobarbital (0.14 mg/kg body weight). Eyeballs were enucleated, and retinas were preserved for subsequent morphological and biochemical analysis.

### 2.5. Assessment of Retinal Morphology

Enucleated eyeballs were fixed in 10% neutral buffered formalin for 24 h, and this was followed by paraffin embedding. Tissue sections at a thickness of 3 µM were taken at 1 mm from the temporal edge of the optic disc and were subjected to hematoxylin and eosin (H&E) staining. The stained retinal sections were examined by two independent observers under a light microscope at 20× magnifications (Olympus IX8, Olympus Corporation, Tokyo, Japan). Microphotographs of 5 randomly selected areas from each section were saved using imaging software (NIS-Elements Basic Research, version 4.30, Nikon Instrument Inc.,Tokyo, Japan). The morphometric measurements were done using Image J software (Image J 1.31, National Institutes of Health, Bethesda, MD, USA). The measurements included: 1) thickness between outer and inner limiting membranes (ILM-OLM), 2) thickness of the inner nuclear layer (INL), 3) thickness of the outer nuclear layer (ONL), and 4) the number of cell nuclei per mm^2^ area of ganglion cell layer (GCL). The average of the measurements by two observers was used for analysis.

### 2.6. Assessment of Retinal Cell Apoptosis with Terminal Transferase dUTP Nick End Labeling (TUNEL Staining)

Apoptotic retinal cells in the GCL were detected using a fluorescence TdT FragEL DNA fragmentation detection kit (Merck Millipore, Beijing, China). The 3 µM tissue sections were placed on poly-lysine coated slides and were deparaffinized using xylene and serial concentrations of ethanol. Antigen retrieval was performed by treating sections with proteinase K (1:100 in 10 mM Tris, pH 8, room temperature) for 20 min, followed by washing with tris-buffered saline (TBS). After rinsing, sections were incubated with terminal deoxynucleotidyl transferase (TdT) equilibration buffer (200 mM potassium cacodylate pH 6.6, 25 mM Tris-HCl pH6.6, 0.2 mM dithiothreitol, 0.25 mg/mL bovine serum albumin (BSA), 2.5 mM cobalt chloride) for 20 min at room temperature. This was followed by incubation with a mixture of labeling reaction mix containing fluorescein and TdT enzyme, overnight at room temperature. After incubation, the sections were washed and were mounted with Fluorescence-FragEL™ mounting media. For positive control, tissue sections were incubated with DNase I (1 µg/µL), followed by incubation with TdT, whereas, for the negative control, the sections were incubated with labeling reaction mix that did not contain TdT enzyme.

Sections were then viewed under a fluorescence microscope (BX5 Fluorescence Trinocular Microscope, Olympus, Tokyo, Japan) at 20× magnifications. The population of retinal cells was visualized with DAPI filter at 330 nm, whereas the labeled nuclei were observed using standard fluorescence filter at 465 nm. Six areas in the GCL were randomly selected in each section, and TUNEL-positive cells were counted using Image J software. The number of apoptotic cells was expressed as the number of cells/mm^2^ area of GCL.

### 2.7. Estimation of Retinal Vascular Endothelial Growth Factors (VEGF) Expression

Retinal VEGF expression was determined using a commercially available ELISA kit (FineTest, Wuhan, China). Isolated retinas were rinsed with ice-cold phosphate buffer saline (0.01 M PBS, pH 7.4) and then were homogenized in radio-immunoprecipitation assay (RIPA; Thermo Scientific, Rockford, IL, USA) buffer with a protease inhibitor in a ratio of 1 mg of retinal weight to 10 µL of RIPA buffer. Homogenized samples were centrifuged at 1600× *g* at 4 °C for 10 min, and the supernatant was collected for analysis.

A hundred microliter of supernatant was added to wells pre-coated with an antibody specific to VEGF, and the incubation was done for 90 min at 37 °C. Next, biotin-detection antibody and horseradish peroxidase (HRP) conjugate were added, followed by incubation for 60 min and 30 min, respectively. The solution in the wells was aspirated, and each well was washed with wash buffer (10 mM phosphate buffer pH 7.4, 150 mM NaCl, 0.05% Tween 20) for five times. 3,3′,5,5′-tetramethylbenzidine (TMB) substrate was then added, and the incubation was done for 20 min at 37 °C. The reaction was stopped by adding sulfuric acid (0.16 M), and the absorbance was read at 450 nm using a microplate reader (Victor X5™, Perkin Elmer, Waltham, MA, USA).

### 2.8. Statistics

The data were presented as mean ± SD. The statistical comparison among experimental groups was done using one-way ANOVA with the posthoc Bonferroni test. The *p*-value of <0.05 was considered significant.

## 3. Result

### 3.1. Effect of TRF on Body Weight

**Figure 1:**
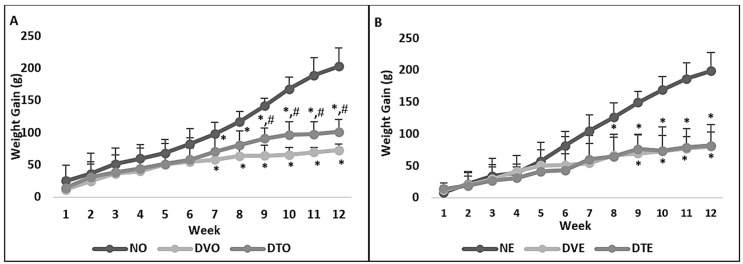
Weight gain (grams) among various groups of rats that received treatment (**A**) orally and (**B**) topically over 12 weeks of the experimental period. NO: Normal rats with oral vehicle treatment, DVO: Diabetic rats with oral vehicle treatment, DTO: Diabetic rats with oral TRF treatment, *n* = 6, * *p* < 0.05 versus NO or NE; # *p* < 0.05 versus DVO. NE: Normal rats with topical vehicle treatment, DVE: Diabetic rats with topical vehicle treatment, DTE: Diabetic rats with topical TRF treatment, *n* = 6.

The weight gain was significantly lower in diabetic rats compared to corresponding normal control rats starting from week 7 to 8 post-STZ-injection (*p* < 0.001). DTO rats showed significantly greater weight gain compared to DVO from week 7 post-STZ-induction until the end of the experimental period (*p* < 0.05), whereas DTE showed no significant difference in body weight compared to DVE at any time point (Figure 1).

### 3.2. Effect of TRF on Blood Glucose Level

**Figure 2:**
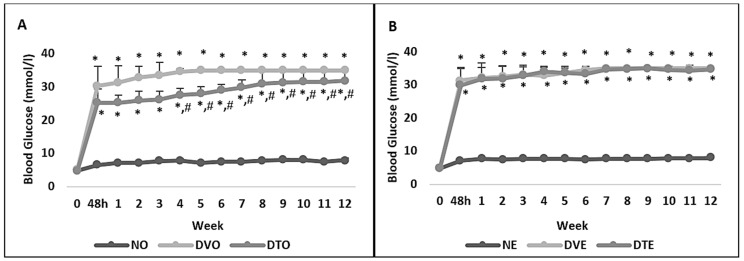
Effect of the tocotrienol-rich fraction (TRF) on blood glucose levels (mmol/L) in rats with streptozotocin (STZ)-induced diabetes. Rats were treated by (**A**) oral and (**B**) topical routes over 12 weeks of the experimental period. NO: Normal rats with oral vehicle treatment, DVO: Diabetic rats with oral vehicle treatment, DTO: Diabetic rats with oral TRF treatment, *n* = 6, * *p* < 0.05 versus NO or NE; # *p* < 0.05 versus DVO. NE: Normal rats with topical vehicle treatment, DVE: Diabetic rats with topical vehicle treatment, DTE: Diabetic rats with topical TRF treatment, *n* = 6.

Diabetic rats showed higher blood glucose levels compared to corresponding normal control rats, starting from 48 h post-STZ-injection until the end of the experimental period (*p* < 0.001). However, the blood glucose level in DTO rats was significantly lower compared to the corresponding control group starting from week 4 post-STZ-injection until the end of the experimental period (*p* < 0.05) (Figure 2). Notably, the blood glucose level in DTO, although, was lower than in DVO, remained significantly greater than NO. Similar observation was not made in the topical treatment group.

**Figure 3:**
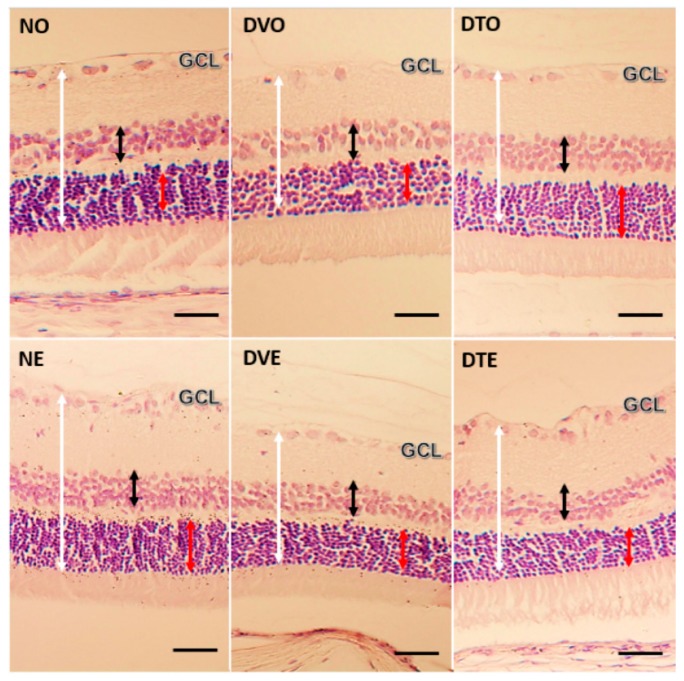
Microphotograph of H&E stained retinal sections from various groups of animals, showing the effect of TRF on retinal layer thickness (magnification 20×). White arrow: Retinal thickness from the inner limiting membrane (ILM) to outer limiting membrane (OLM); Black arrow: Thickness of inner nuclear layer (INL); Red arrow: Thickness of outer nuclear layer (ONL); NO: Normal rats with oral vehicle treatment, DVO: Diabetic rats with oral vehicle treatment, DTO: Diabetic rats with oral TRF treatment. NE: Normal rats with topical vehicle treatment, DVE: Diabetic rats with topical vehicle treatment, DTE: Diabetic rats with topical TRF treatment. (Scale bar: 50 µm).

**Figure 4:**
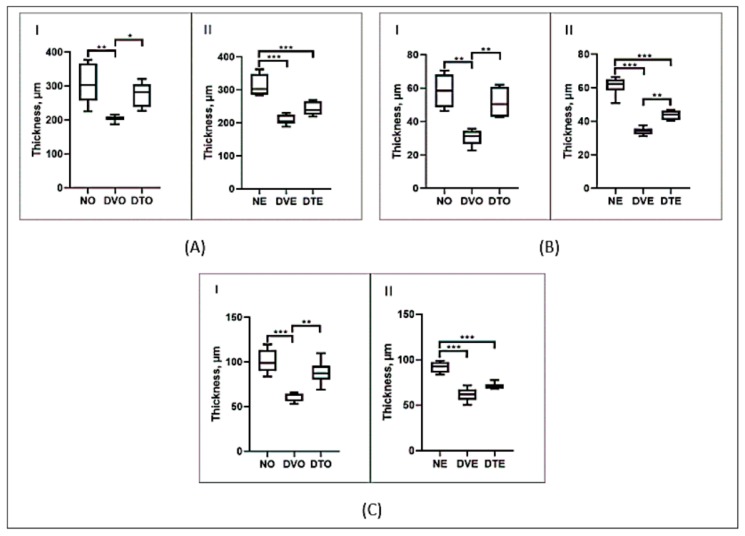
Effect of TRF on the thickness of retina from (**A**) ILM to OLM, (**B**) INL, and (**C**) ONL in rats with STZ-induced diabetes is presented using box and whisker plots. Boxes show median, lower quartile, and upper quartile, whereas whiskers show the data variability outside the upper and lower quartiles. Rats were treated by (**I**) oral and (**II**) topical routes over 12 weeks of the experimental period. NO: Normal rats with oral vehicle treatment, DVO: Diabetic rats with oral vehicle treatment, DTO: Diabetic rats with oral TRF treatment. NE: Normal rats with topical vehicle treatment, DVE: Diabetic rats with topical vehicle treatment, DTE: Diabetic rats with topical TRF treatment. *n* = 6, * *p* < 0.05; ** *p* < 0.01; *** *p* < 0.001.

The retinal layer thickness and cell count measurements were done using H&E stained retinal sections (Figure 3). The thickness of all retinal layers was significantly lesser in diabetic rats compared to normal control rats. However, in the oral TRF-treated group, the thickness of ILM-OLM, INL, and ONL was greater by 1.35-, 1.73-, and 1.44-folds, respectively, compared to the corresponding diabetic control group (*p* < 0.05, *p* < 0.01, and *p* < 0.001, respectively). In the topical TRF-treated group, the thickness of INL but not ILM-OLM was greater by 1.28-folds compared to the corresponding diabetic control group (*p* < 0.01) (Figure 4).

**Figure 5:**
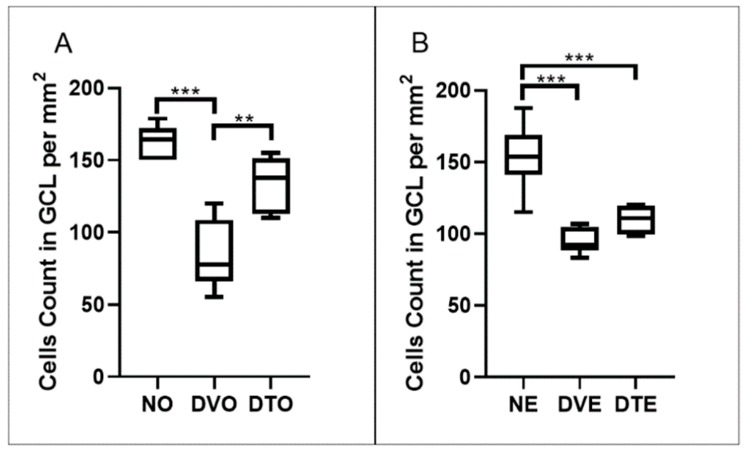
Effect of TRF on the retinal cells count per mm^2^ area of ganglion cell later in rats with STZ-induced diabetes is presented using box and whisker plots. Boxes show median, lower quartile, and upper quartile, whereas whiskers show the data variability outside the upper and lower quartiles. Rats were treated by (**A**) oral and (**B**) topical routes over 12 weeks of the experimental period. NO: Normal rats with oral vehicle treatment, DVO: Diabetic rats with oral vehicle treatment, DTO: Diabetic rats with oral TRF treatment. NE: Normal rats with topical vehicle treatment, DVE: Diabetic rats with topical vehicle treatment, DTE: Diabetic rats with topical TRF treatment, *n* = 6; ** *p* < 0.01; *** *p* < 0.001.

The retinal cell counts in GCL were significantly lower in diabetic control rats compared to corresponding normal control rats (*p* < 0.001). Greater retinal cell counts were observed in DTO compared to the corresponding diabetic control group (*p* < 0.01, respectively). However, no significant difference was seen in the retinal cell counts in DTE compared to the corresponding diabetic control group despite a 1.26-fold difference between these two groups (Figure 5).

### 3.3. Effect of TRF on Retinal Cells Apoptosis

**Figure 6:**
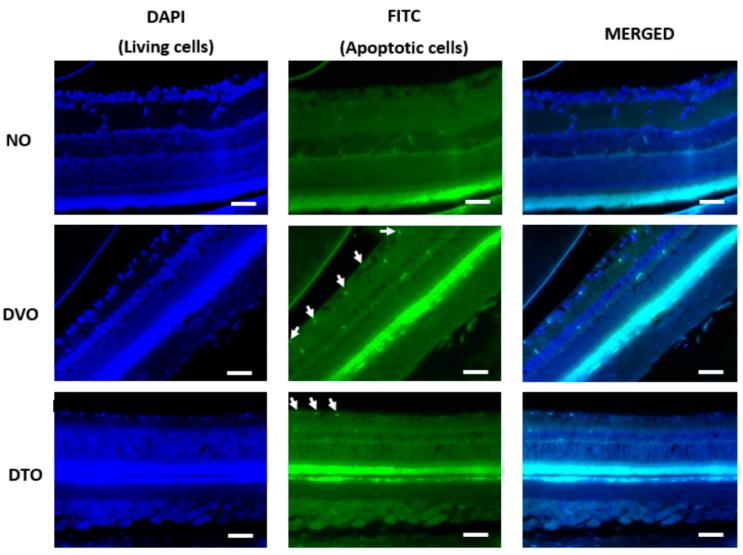
Microphotograph of TUNEL-stained retinal sections, showing the effect of oral TRF on the retinal cell apoptosis (magnification 20×). White arrow: TUNEL positive cells, Left: DAPI; Middle: FITC; Right: DAPI + FITC (Merged). NO: Normal rats with oral vehicle treatment, DVO: Diabetic rats with oral vehicle treatment, DTO: Diabetic rats with oral TRF treatment, GCL: Ganglion cell layer. (Scale bar: 100 µm).

**Figure 7:**
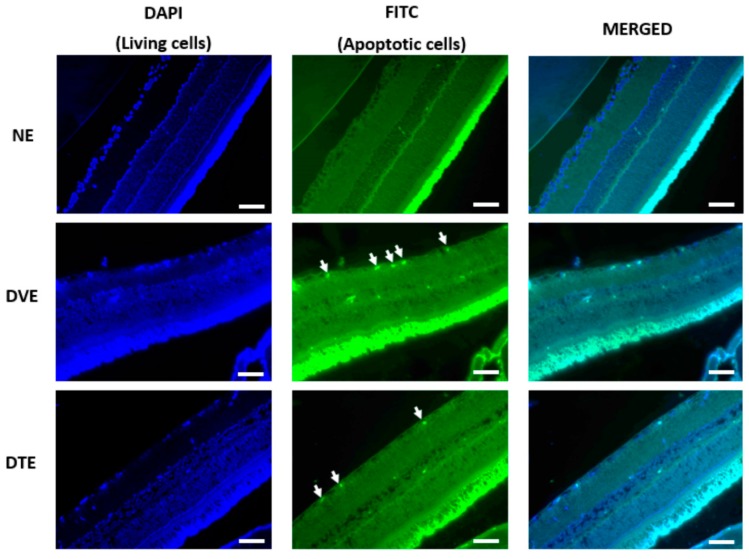
Microphotograph of TUNEL-stained retinal sections, showing the effect of topical TRF on the retinal cell apoptosis (magnification 20×). White arrow: TUNEL apoptotic cells, Left: DAPI; Middle: FITC; Right: DAPI + FITC (Merged). NO: Normal rats with oral vehicle treatment, DVO: Diabetic rats with oral vehicle treatment, DTO: Diabetic rats with oral TRF treatment, GCL: Ganglion cell layer. (Scale bar: 100 µm).

**Figure 8:**
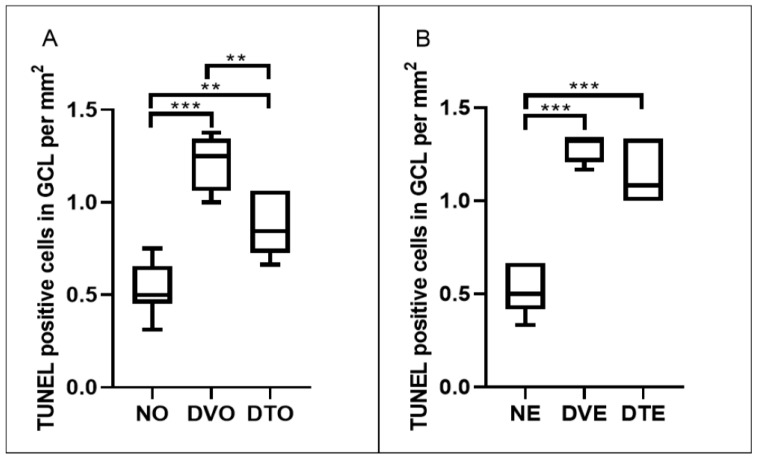
Effect of TRF on the retinal cell apoptosis is presented as TUNEL-positive cells per mm^2^ area of a ganglion cell layer in rats with STZ-induced diabetes. Data is presented using box and whisker plots. Boxes show median, lower quartile, and upper quartile, whereas whiskers show the data variability outside the upper and lower quartiles. Rats were treated by (**A**) oral and (**B**) topical routes over 12 weeks of the experimental period. NO: Normal rats with oral vehicle treatment, DVO: Diabetic rats with oral vehicle treatment, DTO: Diabetic rats with oral TRF treatment. NE: Normal rats with topical vehicle treatment, DVE: Diabetic rats with topical vehicle treatment, DTE: Diabetic rats with topical TRF treatment, *n* = 6; ** *p* < 0.01; *** *p* < 0.001.

In order to determine if the changes observed in the number of cell nuclei in GCL could be due to neuronal apoptosis in this layer, retinal sections were subjected to TUNEL staining. TUNEL-positive retinal cell count was determined, which indicated the extent of retinal cell apoptosis. Some of the sections, such as in NO group, showed a rather diffuse DAPI staining, which might indicate damage to photoreceptors. However, this could not be confirmed as we did not use photoreceptors’ specific markers to determine the involvement of photoreceptors in this study. Since the focus in this study was on the neuronal loss in the GCL, we did not take into account any FITC staining in other layers of the retina. Significantly greater TUNEL-positive retinal cell count was observed in diabetic control compared to corresponding normal control rats (*p* < 0.001). A significantly lesser number of apoptotic cells was observed in the GCL of DTO compared to the corresponding diabetic control group (*p* < 0.01, respectively). However, in DTE, the apoptotic cell count remained comparable to the corresponding diabetic control group (*p* = 0.171) (Figure 6, Figure 7 and Figure 8).

### 3.4. Effect of TRF on Retinal VEGF Expression

**Figure 9:**
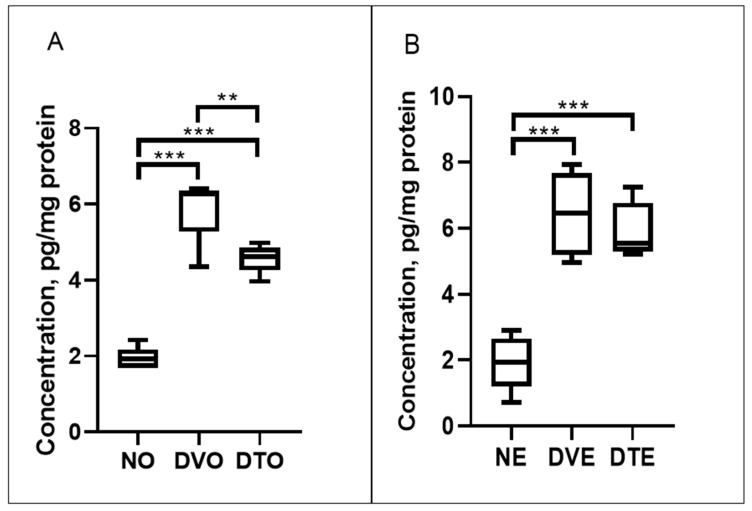
Effect of TRF on the retinal vascular endothelial growth factor (VEGF) expression in rats with STZ-induced diabetes is presented using box and whisker plots. Boxes show median, lower quartile, and upper quartile, whereas whiskers show the data variability outside the upper and lower quartiles. Rats were treated by (**A**) oral and (**B**) topical routes over 12 weeks of experimental period NO: Normal rats with oral vehicle treatment, DVO: Diabetic rats with oral vehicle treatment, DTO: Diabetic rats with oral TRF treatment. NE: Normal rats with topical vehicle treatment, DVE: Diabetic rats with topical vehicle treatment, DTE: Diabetic rats with topical TRF treatment, *n* = 6; ** *p* < 0.01; *** *p* < 0.001.

The VEGF expression in diabetic control rats was significantly greater compared to corresponding normal control rats (*p* < 0.01). VEGF expression in DTO was 1.29-folds lower compared to DVO (*p* < 0.05), whereas DTE did not show a significant difference from the corresponding vehicle-treated diabetic control rats (Figure 9).

## 4. Discussion

The current study, for the first time, demonstrated the effect of TRF against STZ-induced DR in rats. The effect was particularly prominent in rats treated orally with TRF, which showed significantly greater thickness of various layers of the retina and significantly lower retinal cell apoptosis and retinal VEGF levels compared to corresponding controls. Similar effects were not observed in rats treated topically with TRF.

Additionally, in the rats treated with TRF orally, body weight gain was significantly greater than that in the diabetic rats treated with vehicle orally. The greater weight gain in oral TRF-treated rats correlated with the significantly lower blood glucose level in this group of rats compared to corresponding diabetic controls. In accordance with these findings in the current study, other studies that have investigated the effect of palm oil-derived TRF against vascular and pancreatic damage secondary to diabetes mellitus in rats have also made similar observations [20,22]. On the contrary, the topically administered TRF did not affect the body weight and blood glucose level in diabetic rats. This lack of effect of topical TRF on body weight and blood sugar might possibly be attributed to the minimal amount of TRF entering the systemic circulation after topical application.

Indicators of retinal degeneration in DR include reduction of retinal layer thickness (RLT) and retinal cell count and an increase in retinal cell apoptosis [29]. DR manifests clinically with microvascular changes; however, studies have shown that disruption of the neurovascular unit occurs early in the disease process and leads to neurodegeneration, which includes reduced neuronal functions and neural cell apoptosis. Hyperglycemia-induced metabolic dysregulation, as well as oxidative stress, are also important factors that directly contribute to the development of neurodegeneration in DR [30]. In fact, the neurodegeneration in DR may occur even before overt microangiopathic changes [31,32]. The retinal ganglion cells, amacrine cells, as well as photoreceptors, undergo apoptosis, leading to structural changes in the retina and thinning of its various layers [31]. Therefore, at an early stage of DR, reduced RLT may be an important morphological change, indicating neurodegeneration. In fact, such changes in diabetic retinas have been detected by optical coherence tomography (OCT) [33]. In accordance with these observations, in the current study, we observed greater retinal cell apoptosis, consequently causing significant thinning of various layers of the retina in STZ-induced diabetic rats compared to normal rats. In accordance with our findings, other studies have also shown that the induction of diabetes in rats results in the reduced thickness of various retinal layers. Zhang et al. [34] showed that the number of neurons in the GCL, total RLT, and retinal nerve fiber layer thickness significantly reduced after induction of diabetes for 20 weeks in rats. Ali et al. [35] also showed marked areas of cellular dropout in the GCL and reduction of the thickness of the GCL after rats were diabetic for 7 weeks. Similarly, in another study, rats have been shown to have a significant reduction in the thickness of various layers of the retina as well as entire retina using OCT after 30 days of diabetes [36]. One of the studies, however, has shown that the induction of diabetes in rats for a period of 10 weeks results in increased RLT. The reason for these differences remain unclear; however, it is noticeable that, in this study, the blood sugar level of rats has ranged between 14–28 mmol/L, whereas, in our study, the blood sugar levels were consistently close to or above 30 mmol/L. Therefore, a difference in the level of hyperglycemia may have resulted in the differences observed in RLT [37].

Several studies have shown that reduction in retinal layer thickness commonly correlates with a reduction in retinal cell nuclei count and an increase in retinal cell apoptosis, particularly in the GCL [38,39,40], as was observed in the current study. Treatment with antioxidants has been shown to decrease neurodegenerative changes in DR by scavenging free radicals and maintaining endogenous antioxidant defenses [41,42,43]. High retinal oxidative stress in the hyperglycemic environment is suggested to be due to activation of polyol pathway, hexosamine pathway, receptor of advanced glycation end-product (rAGE), protein kinase C (PKC) pathway, and inflammatory cascade [19]. In the current study, increased RLT and retinal cell count with a reduction in retinal cell apoptosis among oral TRF-treated diabetic rats might be attributed to its ability to reduce blood sugar level as well as antioxidant properties. TRF is a potent antioxidant [44], and, additionally, it has been shown to have a direct anti-apoptotic effect against neuronal cells’ apoptosis due to glutamate excitotoxicity [45]. Since excitotoxicity is known to be involved in diabetes-induced retinal neuron loss [46], this direct anti-apoptotic activity of TRF may also underlie its protective effect against changes in retinal morphology in diabetic rats.

The breakdown of the blood-retinal barrier (BRB) and vaso-regression are the early events in DR [47]. These changes create a hypoxic environment in the retina, leading to angiogenesis as a tissue response. However, the newly formed vascular network is non-functional, creating a vicious cycle of hypoxia and more angiogenesis [48]. One of the important proteins that promote angiogenesis is VEGF. Higher retinal expression of VEGF has long been associated with the progression of DR [49]. Accordingly, in the current study, retinal VEGF expression was significantly greater in diabetic rats compared to normal control rats. However, we observed significantly lower retinal VEGF expression in diabetic rats treated with TRF orally, which may or may not be secondary to reduced blood sugar levels in this group of rats as previous studies have also shown that TRF reduces VEGF expression and produces an anti-angiogenic effect against tumor angiogenesis [50]. Importantly, studies have also shown that VEGF may act as a neurotrophic factor and thereby may play a role in maintaining and preserving the neuronal functions [51,52]. Hence, the neuroprotective effects of TRF, as observed in the current study, may be secondary to its effects on VEGF.

It is notable that the effects of TRF on VEGF expression as well as retinal morphology were evident in the oral treatment group but not in the topical treatment group. This might be attributed to a significant lowering of blood sugar by TRF and thereby reducing its consequences, such as microvascular damage and oxidative stress, in the orally treated group but not in the topical treatment group. However, it is important to note that the blood sugar level in rats treated orally with TRF remained significantly greater than normal control throughout the experimental period. Hence, it is likely that factors other than a reduction in blood glucose level contribute to the effect of TRF on VEGF expression and retinal morphology.

In accordance with the findings of the current study, the beneficial effects of oral TRF supplementation have earlier been reported in animal models of brain diseases, such as Alzheimer’s disease [53] and cerebral hypoperfusion [54]. The benefits of TRF in these conditions seem to indicate its ability to cross the blood-brain barrier (BBB). As the blood-retinal barrier (BRB) has similarities with BBB [55], it is expected that orally administered TRF can also penetrate BRB and exert its effect on the retina. Relative lack of effect of topical TRF observed in this study might be attributed to its poor penetration through multiple anatomical and physiological barriers to reach retina [56,57]. In this study, although the topical TRF was used in a microemulsion formulation, which has been shown to have an effect against cataract, an anterior segment disease of the eye [26,28], it is unlikely to reach posterior segment in sufficient concentrations after topical application. In fact, according to our previous studies [26,28], in the current study as well, we observed delay in the development and progression of cataract in both oral and topical TRF-treated groups compared to respective vehicle-treated groups. In the oral TRF-treated group, only 10 out of 32 (31.25%) rats developed full-blown cataract at the end of the experimental period compared to 20 out of 32 (62.5%) rats in the corresponding vehicle-treated group. In the topical TRF-treated group, 16 out of 32 (50%) rats developed full-blown cataract at the same time point compared to 21 out of 32 (65.63%) rats in the corresponding vehicle-treated group. Therefore, even in the anterior segment, the bioavailability of TRF seemed to be better after oral administration compared to topical. Perhaps an improved formulation of TRF or different routes of administration, such as intravitreal injection, may enhance the permeation of TRF to the posterior segment of the eye. Nevertheless, the results of the current study provided direction to further research on the in-depth evaluation of the effect of TRF towards oxidative stress, other angiogenic protein, markers of neuronal damage, and apoptosis.

## 5. Conclusions

This study demonstrated the protective effect of TRF against DR in STZ-induced diabetic rats. This effect of TRF was associated with reduced retinal neurodegenerative changes and VEGF expression. TRF administration by oral route showed higher efficacy compared to its topical administration. Further studies are needed to investigate other mechanisms involved in the protective effect of TRF against DR.

## References

[1] Flaxman S.R., Bourne R.R., Resnikoff S., Ackland P., Braithwaite T., Cicinelli M.V., Das A., Jonas J.B., Keeffe J., Kempen J.H. (2017). Global causes of blindness and distance vision impairment 1990–2020: A systematic review and meta-analysis. Lancet Glob. Health.

[2] Yau J.W., Rogers S.L., Kawasaki R., Lamoureux E.L., Kowalski J.W., Bek T., Chen S.-J., Dekker J.M., Fletcher A., Grauslund J. (2012). Global prevalence and major risk factors of diabetic retinopathy. Diabetes Care.

[3] Sayres R., Taly A., Rahimy E., Blumer K., Coz D., Hammel N., Krause J., Narayanaswamy A., Rastegar Z., Wu D. (2019). Using a deep learning algorithm and integrated gradients explanation to assist grading for diabetic retinopathy. Ophthalmology.

[4] Abougalambou S.S.I., Abougalambou A.S. (2015). Risk factors associated with diabetic retinopathy among type 2 diabetes patients at teaching hospital in Malaysia. Diabetes Metab. Syndr. Clin. Res. Rev..

[5] Nanditha A., Ma R.C., Ramachandran A., Snehalatha C., Chan J.C., Chia K.S., Shaw J.E., Zimmet P.Z. (2016). Diabetes in Asia and the Pacific: Implications for the global epidemic. Diabetes Care.

[6] Zheng Y., Ley S.H., Hu F.B. (2018). Global aetiology and epidemiology of type 2 diabetes mellitus and its complications. Nat. Rev. Endocrinol..

[7] Kowluru R.A. (2019). Mitochondrial Stability in Diabetic Retinopathy: Lessons Learned From Epigenetics. Diabetes.

[8] Sohn E.H., van Dijk H.W., Jiao C., Kok P.H., Jeong W., Demirkaya N., Garmager A., Wit F., Kucukevcilioglu M., van Velthoven M.E. (2016). Retinal neurodegeneration may precede microvascular changes characteristic of diabetic retinopathy in diabetes mellitus. Proc. Natl. Acad. Sci. USA.

[9] Barber A.J. (2003). A new view of diabetic retinopathy: A neurodegenerative disease of the eye. Prog. Neuro Psychopharmacol. Biol. Psychiatry.

[10] Bigagli E., Lodovici M. (2019). Circulating Oxidative Stress Biomarkers in Clinical Studies on Type 2 Diabetes and Its Complications. Oxidative Med. Cell. Longev..

[11] Liu X.-F., Zhou D.-D., Xie T., Hao J.-L., Malik T.H., Lu C.-B., Qi J., Pant O.P., Lu C.-W. (2018). The Nrf2 Signaling in Retinal Ganglion Cells under Oxidative Stress in Ocular Neurodegenerative Diseases. Int. J. Biol. Sci..

[12] Behl T., Kotwani A. (2015). Exploring the various aspects of the pathological role of vascular endothelial growth factor (VEGF) in diabetic retinopathy. Pharmacol. Res..

[13] Zhang D., Lv F., Wang G. (2018). Effects of HIF-1α on diabetic retinopathy angiogenesis and VEGF expression. Eur. Rev. Med Pharmacol. Sci..

[14] Peh H.Y., Tan W.D., Liao W., Wong W.F. (2016). Vitamin E therapy beyond cancer: Tocopherol versus tocotrienol. Pharmacol. Ther..

[15] Zainal Z., Abdul Rahim A., Khaza’ai H., Chang S.K. (2019). Effects of Palm Oil Tocotrienol-Rich Fraction (TRF) and Carotenes in Ovalbumin (OVA)-Challenged Asthmatic Brown Norway Rats. Int. J. Mol. Sci..

[16] Niki E., Abe K., Niki E. (2019). Vitamin E: Structure, Properties and Functions. Vitamin E: Chemistry and Nutritional Benefits.

[17] Muid S., Hamid Z., Nawawi H. (2018). Tocotrienol rich fraction supplement reduces oxidative stress in non familial hypercholesterolaemia: Beyond the lipid lowering capability. Int. Food Res. J..

[18] Kowluru R.A., Kennedy A. (2001). Therapeutic potential of anti-oxidants and diabetic retinopathy. Expert Opin. Investig. Drugs.

[19] Kowluru R.A., Mishra M. (2015). Oxidative stress, mitochondrial damage and diabetic retinopathy. Biochim. Biophys. Acta (Bba) Mol. Basis Dis..

[20] Budin S.B., Othman F., Louis S.R., Bakar M.A., Das S., Mohamed J. (2009). The effects of palm oil tocotrienol-rich fraction supplementation on biochemical parameters, oxidative stress and the vascular wall of streptozotocin-induced diabetic rats. Clinics.

[21] Siddiqui S., Khan M.R., Siddiqui W.A. (2010). Comparative hypoglycemic and nephroprotective effects of tocotrienol rich fraction (TRF) from palm oil and rice bran oil against hyperglycemia induced nephropathy in type 1 diabetic rats. Chem. Biol. Interact..

[22] Siti B.B., Khairunnisa’Md Y., Muhd H., Zariyantey A.H., Jamaludin M. (2011). Tocotrienol-rich fraction of palm oil reduced pancreatic damage and oxidative stress in streptozotocin-induced diabetic rats. Aust. J. Basic Appl. Sci..

[23] Lee H., Lim Y. (2018). Tocotrienol-rich fraction supplementation reduces hyperglycemia-induced skeletal muscle damage through regulation of insulin signaling and oxidative stress in type 2 diabetic mice. J. Nutr. Biochem..

[24] De Silva L., Chuah L.H., Meganathan P., Fu J.Y. (2016). Tocotrienol and cancer metastasis. Biofactors.

[25] Aggarwal V., Kashyap D., Sak K., Tuli H.S., Jain A., Chaudhary A., Garg V.K., Sethi G., Yerer M.B. (2019). Molecular mechanisms of action of tocotrienols in cancer: Recent trends and advancements. Int. J. Mol. Sci..

[26] Nasir N.A.A., Agarwal R., Kadir S.H.S.A., Vasudevan S., Tripathy M., Iezhitsa I., Daher A.M., Ibrahim M.I., Ismail N.M. (2017). Reduction of oxidative-nitrosative stress underlies anticataract effect of topically applied tocotrienol in streptozotocin-induced diabetic rats. PLoS ONE.

[27] Mohamed W.M., Sayeed S., Saxena A.K., Oothuman P. (2018). Oxidative stress status and neuroprotection of tocotrienols in chronic cerebral hypoperfusion-induced neurodegeneration rat animal model. Int. J. Nutr. Pharmacol. Neurol. Dis..

[28] Nasir N.A.A., Agarwal R., Vasudevan S., Tripathy M., Alyautdin R., Ismail N.M. (2014). Effects of topically applied tocotrienol on cataractogenesis and lens redox status in galactosemic rats. Mol. Vis..

[29] Van Dijk H.W., Verbraak F.D., Kok P.H., Stehouwer M., Garvin M.K., Sonka M., DeVries J.H., Schlingemann R.O., Abramoff M.D. (2012). Early neurodegeneration in the retina of type 2 diabetic patients. Investig. Ophthalmol. Vis. Sci..

[30] Ola M., Nawaz M., Khan H., Alhomida A. (2013). Neurodegeneration and neuroprotection in diabetic retinopathy. Int. J. Mol. Sci..

[31] Simó R., Stitt A.W., Gardner T.W. (2018). Neurodegeneration in diabetic retinopathy: Does it really matter?. Diabetologia.

[32] Garcia-Ramírez M., Hernández C., Villarroel M., Canals F., Alonso M., Fortuny R., Masmiquel L., Navarro A., García-Arumí J., Simó R. (2009). Interphotoreceptor retinoid-binding protein (IRBP) is downregulated at early stages of diabetic retinopathy. Diabetologia.

[33] Jiang J., Liu Y., Chen Y., Ma B., Qian Y., Zhang Z., Zhu D., Wang Z., Xu X. (2018). Analysis of changes in retinal thickness in type 2 diabetes without diabetic retinopathy. J. Diabetes Res..

[34] Zhang X., Peng L., Dai Y., Sheng X., Chen S., Xie Q. (2020). Effects of Coconut Water on Retina in Diabetic Rats. Evid. Based Complement. Altern. Med..

[35] Ali S.A., Zaitone S.A., Dessouki A.A., Ali A.A. (2019). Pregabalin affords retinal neuroprotection in diabetic rats: Suppression of retinal glutamate, microglia cell expression and apoptotic cell death. Exp. Eye Res..

[36] He M., Long P., Guo L., Zhang M., Wang S., He H. (2019). Fushiming capsule attenuates diabetic rat retina damage via antioxidation and anti-inflammation. Evid. Based Complement. Altern. Med..

[37] Xi G., Wai C., Clemmons D. (2019). Inhibition of Aberrant IGF-I Signaling in Diabetic Male Rat Retina Prevents and Reverses Changes of Diabetic Retinopathy. J. Diabetes Res..

[38] Arfuzir N., Lambuk L., Jafri A., Agarwal R., Iezhitsa I., Sidek S., Agarwal P., Bakar N., Kutty M., Yusof A.M. (2016). Protective effect of magnesium acetyltaurate against endothelin-induced retinal and optic nerve injury. Neuroscience.

[39] Lambuk L., Jafri A.J.A., Arfuzir N.N.N., Iezhitsa I., Agarwal R., Rozali K.N.B., Agarwal P., Bakar N.S., Kutty M.K., Yusof A.P.M. (2017). Neuroprotective effect of magnesium acetyltaurate against NMDA-induced excitotoxicity in rat retina. Neurotox. Res..

[40] Spaide R.F. (2019). Measurable Aspects Of The Retinal Neurovascular Unit In Diabetes, Glaucoma And Controls. Am. J. Ophthalmol..

[41] Ibrahim Al-Dosary D.S., Alhomida A.S., Ola M. (2017). Protective effects of dietary flavonoids in diabetic induced retinal neurodegeneration. Curr. Drug Targets.

[42] Rohowetz L., Kraus J., Koulen P. (2018). Reactive oxygen species-mediated damage of retinal neurons: Drug development targets for therapies of chronic neurodegeneration of the retina. Int. J. Mol. Sci..

[43] Nawaz M.I., Alhomida A.S., Ola M.S. (2019). The Potential Beneficial Effects of Curcumin in Diabetic Retinopathy. Curcumin for Neurological and Psychiatric Disorders.

[44] Nor Azman N., Goon J., Abdul Ghani S., Hamid Z., Wan Ngah W. (2018). Comparing Palm Oil, Tocotrienol-Rich Fraction and α-Tocopherol Supplementation on the Antioxidant Levels of Older Adults. Antioxidants.

[45] Selvaraju T.R., Khaza’ai H., Vidyadaran S., Mutalib M.S.A., Vasudevan R. (2014). The neuroprotective effects of tocotrienol rich fraction and alpha tocopherol against glutamate injury in astrocytes. Bosn. J. Basic Med Sci..

[46] Smith S.B. (2002). Diabetic retinopathy and the NMDA receptor. Drug News Perspect..

[47] Bosma E.K., van Noorden C.J., Klaassen I., Schlingemann R.O. (2019). Microvascular Complications in the Eye: Diabetic Retinopathy. Diabetic Nephropathy.

[48] Yamaguchi M., Nakao S., Arita R., Kaizu Y., Arima M., Zhou Y., Kita T., Yoshida S., Kimura K., Isobe T. (2016). Vascular normalization by ROCK inhibitor: Therapeutic potential of Ripasudil (K-115) eye drop in retinal angiogenesis and hypoxia. Investig. Ophthalmol. Vis. Sci..

[49] Rezzola S., Nawaz M.I., Cancarini A., Semeraro F., Presta M. (2019). Vascular Endothelial Growth Factor in the Vitreous of Proliferative Diabetic Retinopathy Patients: Chasing a Hiding Prey?. Diabetes Care.

[50] Husain K., Malafa M.P. (2018). Role of Tocotrienols in Chemosensitization of Cancer. Role of Nutraceuticals in Cancer Chemosensitization.

[51] Nishijima K., Ng Y.-S., Zhong L., Bradley J., Schubert W., Jo N., Akita J., Samuelsson S.J., Robinson G.S., Adamis A.P. (2007). Vascular endothelial growth factor-A is a survival factor for retinal neurons and a critical neuroprotectant during the adaptive response to ischemic injury. Am. J. Pathol..

[52] Saint-Geniez M., Maharaj A.S., Walshe T.E., Tucker B.A., Sekiyama E., Kurihara T., Darland D.C., Young M.J., D’Amore P.A. (2008). Endogenous VEGF is required for visual function: Evidence for a survival role on Müller cells and photoreceptors. PLoS ONE.

[53] Ibrahim N.F., Yanagisawa D., Durani L.W., Hamezah H.S., Damanhuri H.A., Ngah W., Zurinah W., Tsuji M., Kiuchi Y., Ono K. (2017). Tocotrienol-rich fraction modulates amyloid pathology and improves cognitive function in AβPP/PS1 mice. J. Alzheimer’s Dis..

[54] Annaházi A., Mracskó É., Süle Z., Karg E., Penke B., Bari F., Farkas E. (2007). Pre-treatment and post-treatment with α-tocopherol attenuates hippocampal neuronal damage in experimental cerebral hypoperfusion. Eur. J. Pharmacol..

[55] Steuer H., Jaworski A., Elger B., Kaussmann M., Keldenich J., Schneider H., Stoll D., Schlosshauer B. (2005). Functional characterization and comparison of the outer blood–retina barrier and the blood–brain barrier. Investig. Ophthalmol. Vis. Sci..

[56] Sahoo S.K., Dilnawaz F., Krishnakumar S. (2008). Nanotechnology in ocular drug delivery. Drug Discov. Today.

[57] Gaudana R., Ananthula H.K., Parenky A., Mitra A.K. (2010). Ocular drug delivery. Aaps J..

